# Prevalence of color vision deficiency among Saudi dentists and its effect on shade selection: a cross-sectional study

**DOI:** 10.3389/fdmed.2026.1906369

**Published:** 2026-09-16

**Authors:** Mai Almarzouki, Lama Abdullah Khateri, Nada J. Farsi, Mohammed K. Yousef

**Affiliations:** 1 Department of Restorative Dentistry, Faculty of Dentistry, King Abdulaziz University, Jeddah, Saudi Arabia; 2 Division of Restorative Department of Dental, King Abdulaziz Medical City, Ministry of National Guard Health Affairs, Jeddah, Saudi Arabia; 3 Department of Dental Public Health, Faculty of Dentistry, King Abdulaziz University, Jeddah, Saudi Arabia

**Keywords:** color vision deficiency, dental aesthetics, dentists, Ishihara test, shade matching

## Abstract

**Background:**

Accurate shade selection is essential for aesthetic dental restorations and relies on the clinician's color perception. Color vision deficiency (CVD) may impair this ability, yet data on its prevalence and clinical impact among dentists in Saudi Arabia are limited. This study aimed to determine the prevalence of CVD and its association with shade-matching accuracy.

**Methodology:**

This cross-sectional study included 150 dental students, interns, general dentists, and specialists. Color vision was assessed using the Ishihara test and categorized as normal, borderline, or red–green CVD. Shade-matching accuracy was evaluated using the VITA Classical Shade Guide under standardized lighting. Group differences were analysed using the Kruskal–Wallis test with *post-hoc* Wilcoxon rank-sum tests and Holm adjustment.

**Results:**

Normal color vision was observed in 78.7% of participants, 6.0% had red–green CVD, and 15.3% showed borderline findings. Shade-matching accuracy differed significantly across groups (*p* < 0.001), with the highest accuracy in participants with normal color vision and the lowest in those with red–green CVD.

**Conclusion:**

CVD is present among dentists in Saudi Arabia and is associated with reduced shade-matching accuracy. Awareness of color vision status may be important in dental education and clinical practice. Routine color vision screening may therefore be warranted as part of dental education curricula and pre-clinical training programmes to identify clinicians who may benefit from corrective strategies or instrument-assisted shade matching.

## 1 Introduction

Precise shade selection is fundamental to achieving optimal aesthetic outcomes in restorative dentistry, including procedures such as dental fillings, crowns, and veneers. The ability to discern subtle variations in tooth color plays a critical role in the success of these treatments. As patient expectations for natural-looking dental restorations continue to rise, accurate color matching has become a key determinant of patient satisfaction and treatment acceptance (1).

The appearance of natural teeth is a complex phenomenon influenced by multiple factors, including hue, value, chroma, translucency, and light reflection. Achieving restorations that harmoniously integrate with surrounding dentition requires a nuanced understanding of these elements (2, 3). Although advanced shade-matching technologies such as colorimeters and spectrophotometers have been introduced to improve accuracy in restorative dentistry, their widespread clinical use remains limited due to high cost, technical complexity, and practical constraints in everyday dental practice (4, 5). Consequently, visual shade assessment using shade guides remains the most commonly used approach in routine clinical settings (6, 7).

Supporting this observation, Okubo et al. reported no significant difference in shade-matching accuracy between colorimetric devices and human observers (8). Color vision deficiency (CVD) refers to an individual's inability to correctly identify colors, commonly assessed using the Ishihara color vision test (2, 9). Intraoral shade matching relies heavily on the clinician's visual discrimination ability (10). Considerable inter-individual variation exists in color perception, particularly in the ability to detect subtle color differences (11). Given its central role in prosthodontic, restorative, and aesthetic dentistry, impaired color vision may compromise shade selection accuracy compared to dentists with normal color vision (12). Despite this, color vision screening is not routinely incorporated into dental education or professional practice.

Although the prevalence of CVD varies across populations worldwide, limited data are available regarding its prevalence and clinical implications among dental professionals, particularly in Saudi Arabia. Considering the critical role of shade matching in restorative outcomes, understanding the prevalence of CVD among Saudi dentists and its association with shade-matching accuracy is clinically relevant. Therefore, this cross-sectional study aimed to determine the prevalence of color vision deficiency among Saudi dentists and evaluate its effect on shade selection accuracy.

## 2 Materials and methods

### 2.1 Study design and ethical approval

The present study was ethically approved by the “King Abdulaziz University Ethical Committee” on 18/02/2024 under protocol number 277-12-23. Informed consent was taken from all participants, and participation was entirely voluntary. The study comprised two assessment components administered within a single cross-sectional framework: (1) screening for color vision deficiency using the Ishihara test, and (2) evaluation of shade-matching accuracy using the VITA Classical Shade Guide.

### 2.2 Sampling and recruitment

The study included 150 participants comprising dental students in their clinical years (5th year, 6th year, and internship), general dentists, and specialists in restorative dentistry, prosthodontics, pediatric dentistry, and related disciplines. The sample size was calculated using the G*Power software. Based on an estimated prevalence of colour vision deficiency (CVD) of approximately 6% among dental professionals (derived from a pilot regional study), with a confidence level of 95% and a margin of error of 5%, the minimum required sample size was approximately 89 participants. The final sample of 150 participants exceeded this requirement, ensuring adequate statistical power.

Participants were recruited using a convenience sampling method through voluntary participation from dental colleges and clinical settings. Eligible individuals were invited to participate, and those who provided consent were enrolled in the study.

### 2.3 Inclusion and exclusion criteria

Dentists and dental students aged 20–60 years who consented to participate were included in the study. All participants underwent CVD screening using the Ishihara test in a controlled environment. Participants were excluded if they reported any known ocular diseases (such as cataract, glaucoma, or retinal disorders), a history of recent eye surgery, or the use of colored or tinted contact lenses or spectacles at the time of testing. These criteria were applied to minimise confounding factors that could influence color perception.

### 2.4 Questionnaire development

A structured questionnaire was newly developed for this study to collect demographic and professional information relevant to the research objectives. The conceptual framework for the second part of the study, assessing shade-matching ability, was informed by previous literature (13–15). The questionnaire underwent pilot testing before the main study to ensure clarity, relevance, and feasibility. Necessary refinements were made based on pilot feedback.

### 2.5 Color vision assessment (Ishihara test)

Color vision was assessed using the Ishihara color vision test administered digitally through a Google Form. The Ishihara plates were presented under standardized viewing conditions. Participants were instructed to identify the number visible on each plate within three seconds while maintaining a viewing distance of approximately 75 cm. The test was conducted under natural lighting conditions with the screen set to full brightness and without the use of screen filters.

Based on standard Ishihara scoring guidelines, participants were classified as having normal color vision, red–green color vision deficiency, or borderline findings. All assessments were supervised by a single trained and calibrated examiner to ensure consistency in administration and data recording. The absence of formal screen colorimetric calibration was acknowledged as a study limitation.

### 2.6 Shade-matching procedure

Shade-matching ability was assessed using the VITA Classical Shade Guide (VITA Zahnfabrik, Bad Säckingen, Germany). Testing was performed under a standardized light source approximating daylight illumination (5500–6500 K) in a controlled indoor environment to minimize inter-session variability.

Each participant was tested using a complete shade guide (Figure 1) along with five randomly selected shade tabs from a second guide of the same make. The included shade guide image (Figure 1) serves only as an illustrative figure to describe the shade selection assessment. To prevent bias, identifying numbers on all shade tabs were concealed. Participants were instructed to visually match each test tab with the corresponding shade from the complete guide. The same testing sequence was used for all participants, and repeat attempts were not permitted. The total number of correctly matched shades was recorded for each participant, with accuracy scores ranging from 1 to 5.

**Figure 1:**
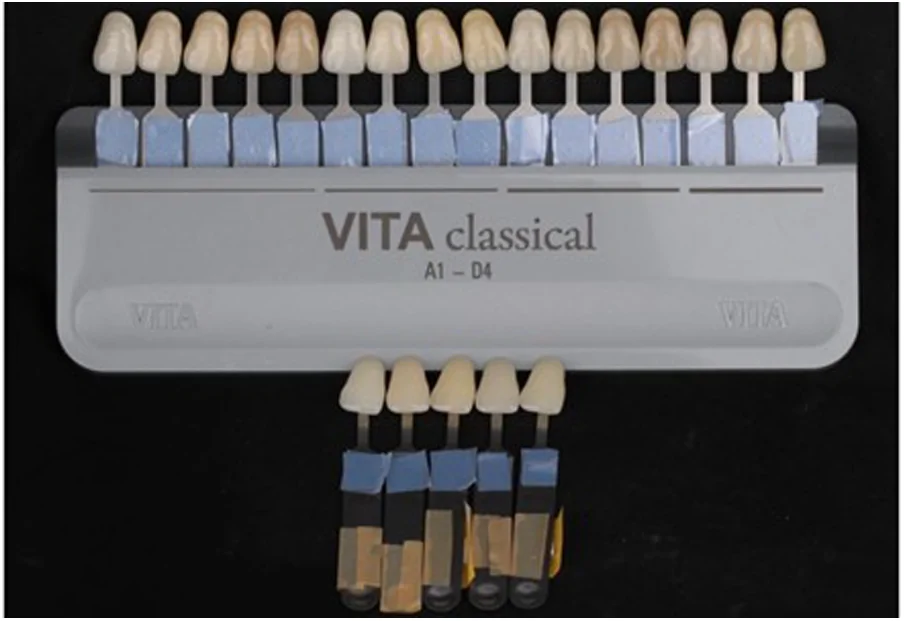
Vita classical shade guide. The included shade guide image serves only as an illustrative figure to describe the shade selection assessment. Photograph of a VITA Classical shade guide with multiple tooth-colored shade tabs organized by the A1–D4 classification system, commonly used for visual shade matching in restorative dentistry.

## 3 Results

### 3.1 Participant characteristics

A total of 150 participants were included in the study. Of these, 84 (56.0%) were female, and 66 (44.0%) were male. The majority of participants were aged 20–30 years (76.7%). Regarding professional status, general dentists constituted the largest professional group, with 45 (30.0%) participants, while restorative dentists represented the largest specialty group, with 18 (42.9%). Most participants practiced in the Makkah region (98.0%). Detailed demographic characteristics are presented in Table 1.

**Table 1:** Participant demographic characteristics (*N* = 150) 367.

Variable	Category	n (%)
Gender	Female	84 (56.0)
	Male	66 (44.0)
Age group	20–30 years	115 (76.7)
	31–40 years	20 (13.3)
	41–50 years	13 (8.7)
	51–60 years	2 (1.3)
Level of education/professional stage	Dental student	27 (18.0)
	Intern	36 (24.0)
	General dentist	45 (30.0)
	Consultant/Specialist	42 (28.0)
Specialty (consultants only)	Restorative dentist	18 (42.9)
	Prosthodontist	12 (28.6)
	Pedodontist	8 (19.0)
	Endodontist	1 (2.4)
	Other	3 (7.1)
Region of practice	Makkah	147 (98.0)
	Eastern Province	1 (0.7)
	Madinah	1 (0.7)
	Tabuk	1 (0.7)

Values are presented as a number (percentage). Percentages may not total 100 due to rounding.

Based on Ishihara plates 1–13, normal color vision was observed in 118 participants (78.7%). Red–green color vision deficiency was identified in 9 participants (6.0%), while 23 participants (15.3%) demonstrated borderline or equivocal findings. The distribution of color vision status is summarized in Table 2.

**Table 2:** Overall color vision status based on Ishihara plates 1–13 (*N* = 150).

Color Vision Status	n	%
Normal	118	78.7
Red-green color vision deficiency	9	6.0
Borderline/equivocal	23	15.3

Color vision status was classified according to standard Ishihara scoring criteria. Borderline findings indicate equivocal responses not meeting criteria for definitive color vision deficiency.

In addition to the diagnostic assessment using Ishihara plates 1–13, responses to selected non-scoring plates were also recorded. Plates 14 and 15, although not included in the diagnostic scoring system, demonstrated concordant response patterns, with normal/total color blindness responses observed in 90.0% (*n* = 135) and 92.0% (*n* = 138) of participants, respectively (Supplementary Table S1).

Responses to plates 16 and 17 showed that the majority of participants demonstrated normal responses (94.0% and 98.7%, respectively), while a small proportion showed deutan or mixed CVD patterns (Supplementary Table S2). Plates 18–23 were excluded due to inconsistent data capture, and plate 24 was non-diagnostic and therefore not included in the analysis.

### 3.2 Shade-matching performance by color vision status

A statistically significant difference in shade-matching accuracy was observed among color vision groups (Kruskal–Wallis test, *p* < 0.001). Participants with normal color vision demonstrated the highest mean shade-matching score (mean ± SD: 3.54 ± 1.07; median [IQR]: 3 [1]). Participants with borderline color vision findings showed lower accuracy (2.96 ± 1.11; median [IQR]: 3 [2]), while those with red–green color vision deficiency had the lowest performance (2.00 ± 1.00; median [IQR]: 1).

Post-hoc pairwise comparisons with Holm adjustment revealed statistically significant differences between participants with normal color vision and those with red–green color vision deficiency (*p* < 0.001), between normal and borderline groups (*p* = 0.0398), and between borderline and red–green color vision deficiency groups (*p* = 0.0301). Shade-matching performance by color vision status is detailed in Table 3.

**Table 3:** Shade-matching performance by color vision status.

Color Vision Status	n	Mean ± SD	Median (IQR)	*P*-value*	Post-hoc pairwise comparisons (Holm-adjusted)
Normal	118	3.54 ± 1.07	3 (1)	<0.001	Normal vs. CVD (*P* < 0.001) Normal vs. border line (*P* = 0.0398) Borderline vs. CVD (*P* = 0.0301).
Borderline	23	2.96 ± 1.11	3 (2)		
Red–green CVD	9	2.00 ± 1.00	2 (1)		

Values are presented as mean ± standard deviation and median (interquartile range). * Differences among groups were analysed using the Kruskal–Wallis test. *Post-hoc* pairwise comparisons were performed using Wilcoxon rank-sum tests with Holm adjustment. Abbreviation: *CVD*, color vision deficiency.

## 4 Discussion

This study evaluated the prevalence of color vision deficiency (CVD) among dental professionals in Saudi Arabia and demonstrated a significant association between color vision status and visual shade-matching accuracy. The findings emphasize the central role of clinician-dependent visual perception in restorative dentistry and highlight an often-overlooked factor that may influence outcomes.

In the present study, red–green color vision deficiency (CVD) was identified in 6.0% of participants, with 78.7% demonstrating normal color vision and 15.3% exhibiting borderline or equivocal findings based on Ishihara plates 1–13. Similar prevalence has been observed among dental students by Khalid et al., where Red–green CVD cases were prevalent amongst male participants (16). Higher prevalence rates have been documented in Nepal (4.0–5.83%) (17), Egypt (4.5–6.9%) (18), and India (4.69%) (12), underscoring the influence of genetic and ethnic diversity across populations. Conversely, lower prevalence was reported in studies from Saudi Arabia (2.1–3.9%) (9), Pakistan (2.0–3.75%) (19), and Malaysia (3.2%) (20). The observed variation in prevalence across regions highlights the importance of contextual interpretation of CVD data. Within clinical and educational settings, these findings support the relevance of routine color vision screening, particularly in disciplines where accurate color discrimination is important to diagnostic accuracy and aesthetic outcomes.

The significant difference (*p* = 0.001) in shade-matching accuracy was observed across the groups. Shade matching is a complex visual task dependent on intact trichromatic vision to discriminate subtle hue, chroma, and value differences (21, 22). Our results, showing a downward performance gradient from normal vision to borderline to red-green CVD, directly align with the physiological basis of color perception (15, 23, 24). Previous studies have also linked CVD to reduced performance in color-related dental tasks, such as detecting gingival inflammation or assessing tooth vitality (5, 10, 25). Our findings add shade matching, a cornerstone of aesthetic dentistry, to the list of clinical skills potentially compromised by CVD.

A key finding of the current study is the inclusion and analysis of participants with borderline or equivocal color vision findings, a group often excluded in binary (normal/defective) classifications. This subclinical group exhibited significantly lower shade-matching accuracy than the normal vision group, though not as poor as the definite CVD group. This suggests a spectrum of color discrimination ability, where even minor deviations from optimal color vision can impact performance. Human color perception exhibits natural inter-individual variation even among those considered “normal” (26, 27). Our findings argue that this range of ability is clinically relevant. Subclinical impairment may not be detected in routine screenings but can still contribute to the high inter-operator variability reported in visual shade matching (5), emphasizing that color competence is not a uniform trait.

Studies have demonstrated limitations of the Ishihara test, as individuals with mild color vision deficiency (CVD) may still correctly identify the primary color (10, 11, 25). Other methods, such as the Nagel anomaloscope test and the Farnsworth–Munsell Hue test, are more accurate but are time-consuming and therefore unsuitable for mass screening (9, 14). Consequently, the Ishihara test was selected, as previous studies have suggested that it is simple, rapid to administer, and sufficiently reliable for large-scale screening of CVD (3, 25, 28). However, to avoid any discrepancies and to get accurate results in the current study, the authors decided to evaluate color deficiencies using 13 plates.

In the dental practices, while instrumental shade matching (spectrophotometers) is available, visual methods remain prevalent due to cost, accessibility, and the need to compare color with surrounding oral structures (7, 18, 29). Instruments provide objective data but cannot fully replicate the integrative visual judgment under variables such as clinical lighting conditions. Borbery carried out a study on 31 dental professionals and found that CVD students had significantly worse color matching potential than the computer-assisted CVD corrector analyser (30). Hence, they mentioned the utilization of the tooth guide trainer to reduce errors in tooth shade selection for CVD practitioners. These findings further support the importance of early screening for color vision deficiencies in dental students, allowing the timely implementation of adaptive training strategies and assistive tools to enhance shade-matching accuracy from the beginning of clinical education.

Evidence from dental education research indicates that color vision deficiency (CVD) may influence shade-matching performance, particularly in hue and chroma discrimination. Studies among dental students and practitioners have demonstrated significantly lower shade-matching accuracy in individuals with CVD compared with those with normal color vision, including reduced correct matching rates (30.7% vs. 63.0%) and poorer color discrimination scores (13% vs. 44%) (11, 28, 31). Other investigations, however, including the present study and prior reports, have observed comparable shade-matching performance between individuals with and without CVD, suggesting contextual and training-related influences (12, 29). Importantly, several studies have shown that targeted visual training and computer-assisted learning tools can improve shade-matching performance under simulated CVD conditions (3, 10, 22, 30). Within dental education and clinical training settings, awareness of individual color vision status may therefore represent a relevant factor influencing visual task performance, particularly where aesthetic judgment is central to patient care.

Several limitations should be considered. The cross-sectional design precludes causal inference. The sample was drawn primarily from a single geographic region and consisted largely of younger professionals (76.7% aged 20–30), which may limit generalisability to the broader dental workforce. The small number of participants with definite red–green CVD (*n* = 9) limited detailed subgroup analysis. In addition, the digital administration of the Ishihara test may differ in luminosity and contrast from printed plates, and the controlled shade-matching task may not fully reflect clinical conditions. The voluntary nature of participation may also have introduced selection bias, and all assessments were conducted by a single examiner, precluding evaluation of inter-examiner reliability.

Despite these limitations, the study benefits from a validated color vision assessment, a standardized shade-matching protocol, controlled testing conditions, and appropriate non-parametric statistical analysis, supporting the reliability of the observed associations.

## 5 Conclusions

This study demonstrates that color vision deficiency is present among dentists in Saudi Arabia and may influence visual shade-matching performance. As visual shade selection remains widely used in clinical practice, awareness of individual color vision status is important within dental education and professional training. Such awareness may support the adoption of adaptive strategies, including visual training and supplementary shade-matching tools, to improve aesthetic outcomes. Future multicentre studies with larger and more diverse samples are needed to further evaluate the relationship between color vision status, training interventions, and clinical performance in restorative dentistry.

## Data Availability

The original contributions presented in the study are included in the article/Supplementary Material, further inquiries can be directed to the corresponding author.
