# Supplementary material for: Prevalence of color vision deficiency among Saudi dentists and its effect on shade selection: a cross-sectional study
Source: Front Dent Med. 2026 Sep 16;7:1906369. doi: 10.3389/fdmed.2026.1906369 (PMC13623585; doi:10.3389/fdmed.2026.1906369)
Supplement: Supplementary file 1 [file Table1.docx]

***Supplementary Material***

Supplementary Tables

**Table S1. Diagnostic patterns for Plates 14–15**

| **Plate** | **Normal/TCB n (%)** | **CVD-pattern n (%)** |
| --- | --- | --- |
| 14 | 135 (90.0) | 15 (10.0) |
| 15 | 138 (92.0) | 12 (8.0) |

**Table S2. Plate 16 and 17 subtype patterns (N = 150)**

| **Category** | **Plate 16 n (%)** | **Plate 17 n (%)** |
| --- | --- | --- |
| **Normal** | 141 (94.0) | 148 (98.7) |
| **Protan pattern** | 0 (0.0) | 0 (0.0) |
| **Deutan pattern** | 1 (0.7) | 2 (1.3) |
| **CVD–other / mixed** | 8 (5.3) | – |
